# Unique Method of Transjugular Intrahepatic Portosystemic Shunt Reduction for Refractory Hepatic Encephalopathy

**DOI:** 10.7759/cureus.18838

**Published:** 2021-10-17

**Authors:** Ayub Khan, Christopher W Bailey

**Affiliations:** 1 Medicine, West Virginia School of Osteopathic Medicine, Lewisburg, USA; 2 Interventional Radiology, Virginia Commonwealth University School of Medicine, Richmond, USA

**Keywords:** transjugular intrahepatic portosystemic shunt, tips, refractory gastrointestinal bleed, decompensated cirrhosis, gastric variceal bleeding, shunt reduction, portal hypertension, tips reduction, hepatic encephalopathy, esophageal varices

## Abstract

We report the case of a 27-year-old female patient with a history of cryptogenic cirrhosis who was admitted to the hospital due to multiple episodes of hematemesis secondary to bleeding esophageal varices. The varices were persistent and refractory to endoscopic intervention, so an emergent transjugular intrahepatic portosystemic shunt (TIPS) was performed by interventional radiology (IR). Months later, the patient returned to the hospital unresponsive with acute intermittent hepatic encephalopathy which required a TIPS reduction by IR. Once the TIPS reduction was performed, the patient’s hepatic encephalopathy declined, and her symptoms improved. Here, we present a unique method of TIPS reduction utilizing a single Viabahn VBX balloon-expandable stent (W. L. Gore & Associates, Inc; Flagstaff, AZ) to constrain the existing TIPS stent.

## Introduction

Patients with a history of cirrhosis suffer a high risk of developing portal hypertension and resultant esophageal varices. Cirrhotic liver resists portal blood flow which then stresses the various portacaval anastomoses, and these veins can often rupture and bleed [1-5]. An accepted treatment for uncontrolled variceal hemorrhage is a transjugular intrahepatic portosystemic shunt (TIPS) procedure that shunts portal blood away from the liver parenchyma by creating an intrahepatic channel between the hepatic vein and a branch of the portal vein to relieve hypertension in the portal system [1,5-9]. The TIPS procedure, however, is sometimes followed by complications such as development of acute hepatic encephalopathy seen in up to 8% of patients, which we present in our case [6-8,10,11]. Current literature outlines treatments such as pharmacological therapy, shunt occlusion, and shunt reduction to manage the hepatic encephalopathy [10-17]. In our report, we present a unique method of reducing the TIPS by constraining the existing stent with a single balloon-expandable stent.

## Case presentation

A 27-year-old female with a history of cryptogenic cirrhosis, idiopathic pancreatitis treated with total pancreatectomy with islet autotransplantation (TPIAT), Billroth II gastroduodenostomy, splenectomy, and secondary diabetes presented to the emergency department (ED) with complaints of four to five days of abdominal pain and multiple episodes of bright red hematemesis. Lab results at the outside hospital prior to ED arrival revealed a significant anemia with a drop in hemoglobin from 9.2 g/dL to 5.6 g/dL. At the ED, the patient had around 1.25 L of hematemesis and became tachycardic and hypotensive. The patient was treated with blood transfusions, intubated for airway protection, and transferred to the ICU.

**Figure 1:**
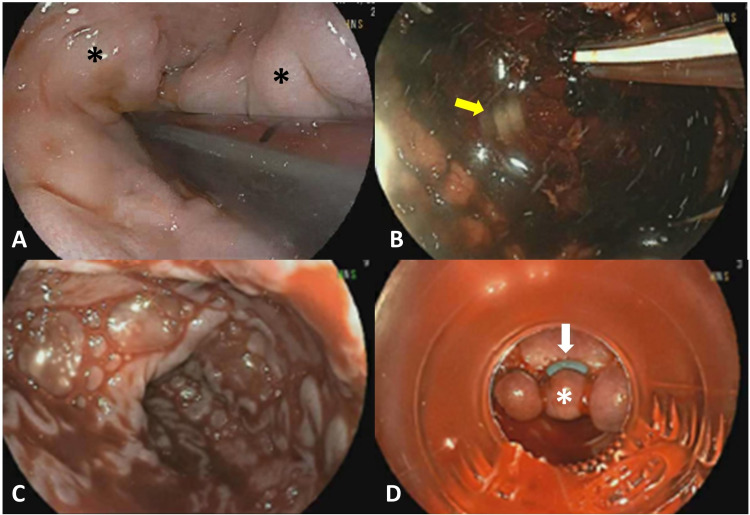
Upper endoscopy #1 and endoscopy #2 Upper endoscopy #1 (A and B) and upper endoscopy #2 (C and D). Image A demonstrates large (>5 mm) varices (*) about the lower third of the esophagus. Hemostatic clip (yellow arrow) was placed at a site of active bleeding at the gastrojejunal anastomosis in this patient status post-Billroth II reconstruction (B). Image C demonstrates continued bleeding about large (>5 mm) varices involving the lower third of the esophagus on repeat upper endoscopy. Banding (white arrow) of an esophageal varix (*) to control bleeding (D).

Gastroenterology was consulted, and prior to the initial endoscopy, the patient was immediately started on an octreotide and pantoprazole infusion. The upper GI endoscopy was technically difficult due to excessive bleeding and revealed large (>5 mm) varices in the lower third of the esophagus with no stigmata of recent bleeding (Figure 1A, 1B). The spurting arterial bleeding source was identified at the gastrojejunal anastomosis (Billroth II reconstruction), and hemostatic clips were placed to stop active bleeding. The patient had repeat active bleeding later that day, and a second endoscopy revealed red blood in the esophagus and large (>5 mm) bleeding esophageal varices (Figure 1C, 1D), which were then banded. Incomplete eradication of the varices was observed by visualization of continued bleeding.

**Figure 2:**
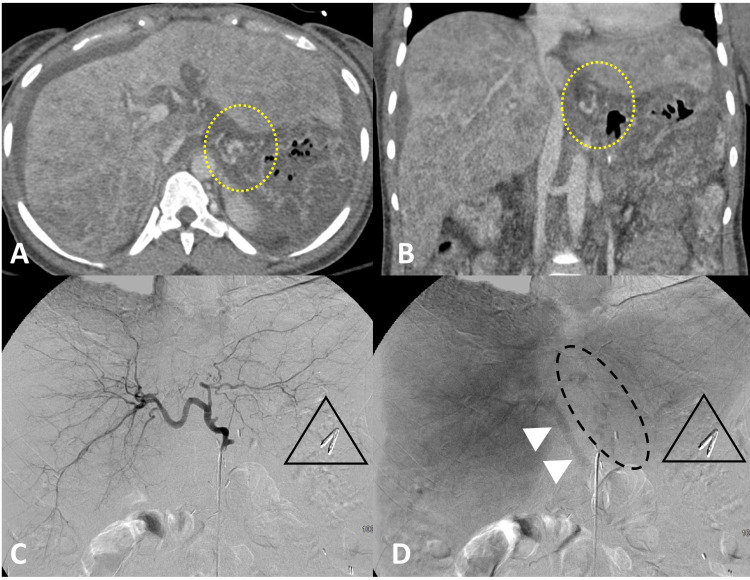
CT imaging of the abdomen and hepatic angiogram Contrast-enhanced CT imaging of the abdomen in the delayed, portal venous phase, 100-second delay, (A and B) demonstrates a nodular/lobulated liver contour with increased heterogeneity throughout the hepatic parenchyma with evidence of sequela of portal hypertension, including gastroesophageal varices (yellow perforated circle). Axial slice (A). Coronal slice (B). Hepatic angiogram images (C and D). Early arterial phase (C) showing no evidence of active arterial bleeding, as well as the presence of endoscopy clips (black triangle). Delayed portal venous phase images from the hepatic angiogram (D) demonstrate patency of the portal vein (white arrowheads) and faintly opacified esophagogastric varices (perforated black oval), as well as presence of endoscopy clips (black triangle). CT: computed tomography.

Helical CT angiography of the abdomen and pelvis was performed after the initial endoscopy and demonstrated distal esophageal varices and cirrhotic liver (Figure 2A, 2B). After the second endoscopy for repeat bleeding, interventional radiology (IR) performed a mesenteric angiogram, which demonstrated no active arterial bleeding. However, on the delayed portovenous phase, small lower esophageal and gastric varices were identified (Figure 2C, 2D).

**Figure 3:**
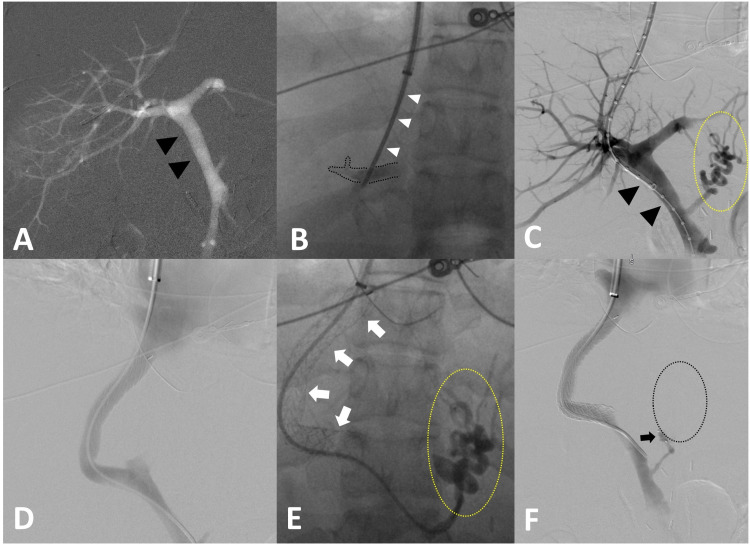
TIPS placement Placement of TIPS using Viatorr stent and embolization of the left gastric vein with an Amplatzer vascular plug (AVP IV). CO_2_ portogram via balloon occlusion catheter in the right hepatic vein (A) showing patent intrahepatic portal branches and the main portal vein (black arrowheads). Image B demonstrates a 16-G Colapinto needle (white arrowheads) transiting across the liver parenchyma into a right intrahepatic portal vein branch (perforated black outline). Digital subtraction portogram illustrating the origin of esophageal varices from the coronary vein (yellow perforated circle) and the main portal vein (black arrowheads) (C). Post-TIPS placement digital subtraction shuntogram (D). In image E, the TIPS stent is shown (white arrows) with contrast opacification of esophageal varices from the coronary vein (yellow perforated circle). Post-Amplatzer vascular plug (AVP IV) embolization of the coronary vein (black arrow) showing absence of esophageal varices on contrast injection (black perforated oval) (F). TIPS: transjugular intrahepatic portosystemic shunt.

Concern for uncontrolled esophageal variceal bleeding led to performance of a TIPS placement using a 10-mm TIPS Viatorr stent (W. L. Gore & Associates, Inc; Flagstaff, AZ) (Figure 3A-3F). Amplatzer vascular plug (AVP IV) (AGA Medical Corporation; Plymouth, MN) embolization of the left gastric vein was also performed. The post-TIPS portosystemic gradient was 8 mmHg (normal range: <12 mmHg). The patient was subsequently discharged after reaching stable condition. On the day of the TIPS procedure, the patient presented with a Model for End-Stage Liver Disease (MELD) score of 21 and Child-Pugh class C. Approximately 10 months prior to the TIPS, a liver biopsy was performed demonstrating cirrhosis, but severity/classification was not mentioned.

Four months later, the patient returned to the ED unresponsive and tachycardic with a Glasgow Coma Scale (GCS) of 6. Her family reported that she had been having mild intermittent hepatic encephalopathy (HE) since the TIPS procedure, which later acutely worsened and prompted the ED visit. She had been using lactulose titrated to have four to five bowel movements per day and rifaximin 550 mg two times per day (BID) to manage encephalopathy. Lab results upon admission revealed venous ammonia of 239 µmol/L (normal range: 11-32 µmol/L), and the patient received a nasogastric (NG) tube for administration of lactulose to mitigate the encephalopathy. Mental status improved with lactulose administration, and ammonia reduced to 84 µmol/L (normal range: 11-32 µmol/L) over three days. A liver vascular ultrasound with Doppler was performed which demonstrated patent TIPS with appropriate directional flow of portal vasculature.

Given the recurrent hepatic encephalopathy refractory to medication post-TIPS, reduction of the TIPS diameter was discussed and agreed upon. Constraining the TIPS diameter was performed by IR. The method of TIPS reduction was unique and utilized a single Viabahn VBX balloon-expandable stent to constrain the existing TIPS stent. The procedure was effective, raising the portosystemic gradient, and on postoperative day 1, the patient demonstrated significant improvement in her symptoms. Six months after the TIPS reduction procedure, the patient was seen in follow-up for multivisceral transplantation given her history of liver cirrhosis and idiopathic pancreatitis treated with TPIAT resulting in secondary diabetes. Since the TIPS reduction procedure, she has had no episodes of rebleeding. A follow-up endoscopy has been ordered, but the patient has not yet scheduled it. Additionally, she has had a few admissions at outside facilities for altered mental status presumably from HE. At least one of these admissions for altered mental status was eventually attributed to hypoglycemia. The patient believes her HE symptoms have worsened after the TIPS reduction procedure, and so she remains on lactulose and rifaximin medications for medical management.

## Discussion

Cirrhosis drastically impairs functioning of the liver and increases the risk of developing intrahepatic portal hypertension and its subsequent complications. In cirrhotic liver, chronic scarring of liver tissue causes an increase in intrahepatic outflow resistance of portal blood flow through the liver. This increased resistance of portal blood flow in addition to the increased venous inflow into the portal system that is observed in cirrhosis cumulatively increases the portal blood pressure [2-5]. Portal hypertension is diagnosed when the hepatic venous pressure gradient (HVPG) between the systemic venous system and the hepatic portal venous system measures >5 mmHg [1,2,5].

To mitigate the portal hypertension, the body shunts up to 90% of portal blood away from the liver and delivers the blood directly to the heart through various anastomoses of the portal and systemic venous systems, such as between the gastric and distal esophageal veins as seen in our patient. The veins present as varices when they become dilated due to persistent hypertension, which can burst when the tension in the venous walls surpasses their elastic limit, resulting in life-threatening hemorrhage [2-5]. Our patient initially presented to the ED with multiple episodes of hematemesis indicating an upper GI bleed caused by an acute rupture of her gastroesophageal varices as confirmed by endoscopy and CT angiography (Figures 1, 2).

Gastroesophageal variceal hemorrhage is the cause of mortality in approximately one-third of patients with cirrhosis, and without appropriate treatment, an estimated 60% of those patients will experience variceal rebleed within one to two years of the initial bleed [3-5]. The established initial treatment for acute variceal bleeding is vasoactive drugs in conjunction with endoscopic therapy [2,7,9]. In our patient, we observed persistent variceal bleeding refractory to treatment with octreotide and band ligation under endoscopy, and this prompted the decision to perform a TIPS procedure, which is the established second-line therapy after unsuccessful endoscopic and pharmacological treatment of variceal bleeding [1,2,4,5,7,9].

TIPS procedure creates an intrahepatic shunt between the portal and hepatic veins, commonly through the use of a covered stent. This shunt allows portal blood flow to bypass the cirrhotic liver and travel directly back to the heart, effectively controlling portal hypertension and diminishing rebleeding rates to about 18% [2,5-8]. Pertinent indications for TIPS procedure that guided our decision making include refractory variceal hemorrhage and secondary prophylaxis of recurrent variceal bleeding. Contraindications for TIPS procedure include fulminant liver failure, heart failure, and refractory HE [6-8]. An important objective for TIPS procedure is to reduce the HVPG <12 mmHg to prevent variceal rebleeding [1,6-8]. Hemodynamic and clinical success was achieved in our TIPS procedure with cessation of variceal bleeding and a post-TIPS HVPG of 8 mmHg [8].

TIPS is considered a second-line therapy because it can cause postprocedural complications such as HE, TIPS stenosis, and puncture of the liver capsule [6-8,11,14]. Our patient returned to the ED several months after TIPS placement, unconscious with a history consistent with recurrent HE that developed after the procedure. HE commonly develops in up to 50% of patients post-TIPS procedure and occurs when there is an accumulation of enteric neurotoxins such as ammonia in the brain, which can then disrupt neural communication and present clinically as behavioral changes, cognitive impairment, and even coma in more severe cases [10,11,15,16]. It is theorized that elevated systemic ammonia levels notably play a key role in the development of HE. In patients post-TIPS, portal blood containing ammonia bypasses the liver, which is normally responsible for detoxifying ammonia [10,11,15]. The TIPS shunts the blood away from the liver parenchyma and into the systemic circulation, allowing undetoxified ammonia to accumulate and gain access to the brain [10,11,15,16].

Currently, accepted treatments for acute HE consistent with current guidelines include noninvasive treatment such as dietary modification and nonabsorbable disaccharides such as lactulose to remove ammonia from the enteric system, in effect lowering systemic ammonia levels. Invasive treatments include liver transplantation and other endovascular treatments such as TIPS shunt reduction and shunt occlusion, which reduce the portal blood volume bypassing the liver through the shunt and increase the portal blood volume entering the liver parenchyma. This effectively increases the amount of ammonia detoxified by the liver and improves the symptoms of HE [10,11,15,18]. Invasive treatments are reserved for up to 8% of patients who experience HE post-TIPS refractory to noninvasive treatment [11,16,17]. History revealed that our patient had been consistently taking lactulose and rifaximin to manage encephalopathy. However, she still experienced recurrent HE, and pharmacological efforts in the ED with lactulose only reduced ammonia levels from 239 µmol/L to 84 µmol/L (normal range: 11-32 µmol/L). This guided our decision to perform a TIPS reduction procedure.

Shunt reduction and shunt occlusion are effective treatments for refractory HE particularly for patients who are not eligible for liver transplantation [10,15]. Shunt reduction methods involve intentionally reducing the diameter of the TIPS lumen, effectively reducing the volume of portal blood flowing through the TIPS while still maintaining shunt patency. Shunt occlusion methods include the use of coil embolization and vascular plugs to fully close the TIPS lumen and eliminate blood flow through the TIPS [10-17]. In a study by Kochar et al. [14], 58% of patients who underwent TIPS modification for refractory HE saw an improvement in HE, with similar improvement results between shunt occlusion and shunt reduction. While both endovascular treatments show effectiveness in treating refractory HE, they are associated with an increased risk of portal hypertension and recurrence of the initial TIPS indication [10-16]. Safety concerns are more prevalent with shunt occlusion methods, and several life-threatening complications have been noted in the literature [10-12,14-16]. Kochar et al. [14] investigated shunt modification outcomes in 38 patients and reported that eight patients in their study died within one week following shunt occlusion. Three of those patients died due to complications associated with the occlusion procedure, and it is suggested that the rest of the deaths resulted from portal hypertensive complications following shunt occlusion [14,16]. In comparison, Schindler et al. [16] investigated predominately shunt reduction (*n* = 18), reporting that no patients suffered procedural related complications and that there was no significant association between shunt reduction and recurrence of variceal bleeding. Similarly, Sarwar et al. [13] reported no recurrence of variceal bleeding following TIPS shunt reduction. Based on the available literature and the author’s opinion, TIPS reduction offered a safer treatment option to improve our patient’s HE while maintaining patency of the TIPS to prevent recurrent variceal bleeding.

**Figure 4:**
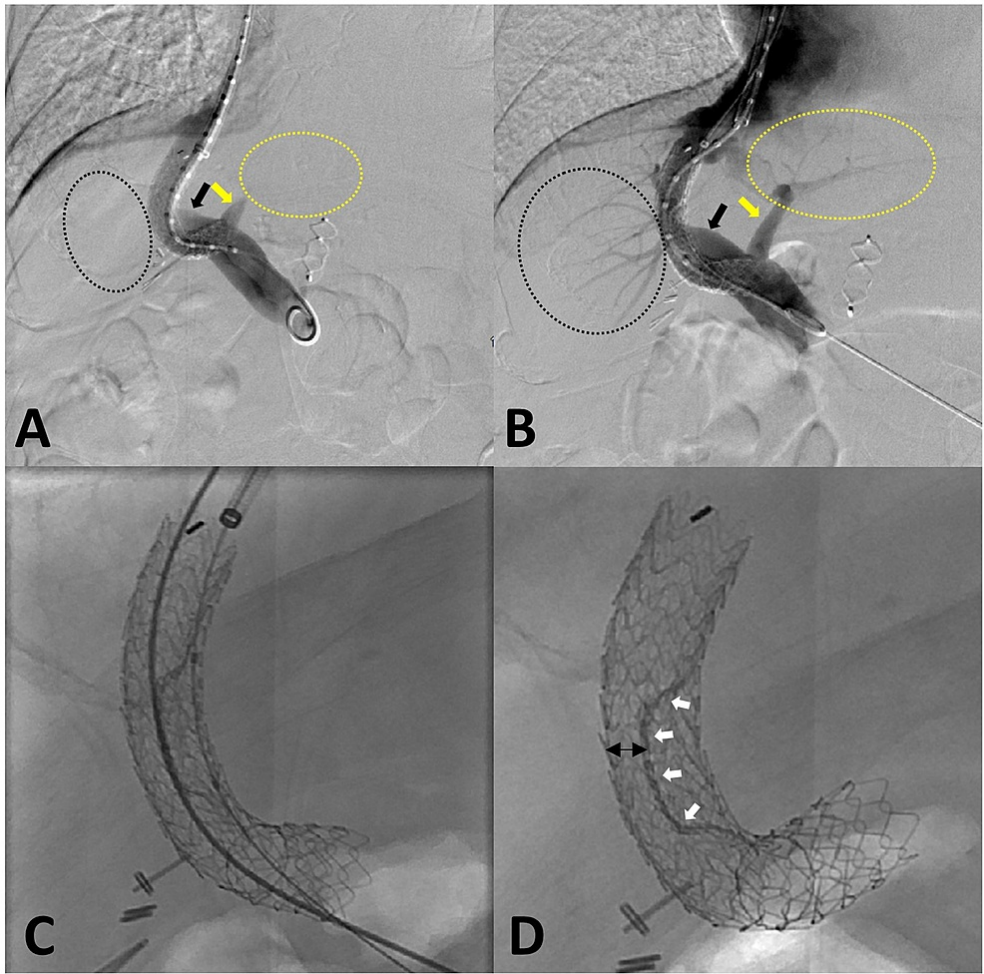
Parallel stent technique for TIPS reduction TIPS reduction, parallel technique. Digital subtraction TIPS shuntogram prior to stent diameter reduction demonstrates a patent TIPS shunt with absence of flow in left intrahepatic portal branches (perforated yellow circle), hepatofugal flow in the left portal vein (yellow arrow), and similar findings of the right portal vein and intrahepatic portal branches (black arrow and perforated black circle) (A). After TIPS reduction, digital subtraction TIPS shuntogram shows a reduced diameter of a patent TIPS shunt with improved hepatopetal flow in left (yellow arrow and perforated yellow circle) and right (black arrow and perforated black circle) portal veins (B). Immediate postdeployment of parallel stents within preexisting TIPS to reduce the diameter using two separate access points (C). Final image of TIPS reduction via parallel technique with reduced diameter of TIPS shunt (black arrows) and the outline of constrained middle (white arrows) (D). TIPS: transjugular intrahepatic portosystemic shunt.

Various techniques for shunt reduction have been discussed in the literature for the treatment of refractory HE. In the past, Forauer and McLean [10,15,19] described a method of shunt reduction by constraining an uncovered Wallstent (Boston Scientific, Natick, MA) inside a balloon-expandable Palmaz stent (P154M; Johnson & Johnson, Warren, NJ) positioned in a parallel fashion within the existing TIPS stent. Another early method of TIPS reduction described constraining the midsegment of an uncovered Wallstent within the TIPS stent with a silk suture [10,15,20]. These methods of TIPS reduction effectively reduced the TIPS lumen diameter and improved the symptoms of HE, but due to the uncovered nature of the Wallstent, they demonstrated limited regulation of shunt blood flow and measurement of changes in HVPG [10,15]. More recently, a parallel stent reduction technique was described by Rowley et al. [12,17], which involved deployment of a balloon-expandable Express SD stent (Boston Scientific, Natick, MA) and a covered Viatorr stent (W. L. Gore & Associates, Inc; Flagstaff, AZ) over two different guidewires in a parallel fashion within the existing TIPS stent. In this method, both stents are expanded simultaneously to create a constrained portion in the middle of the existing TIPS lumen, and a resultant example of this method is demonstrated in Figure 4A-4D. Other common methods of TIPS reduction described in the literature include the use of suture-constrained self-expandable stents and tapered reduction stent grafts [13].

**Figure 5:**
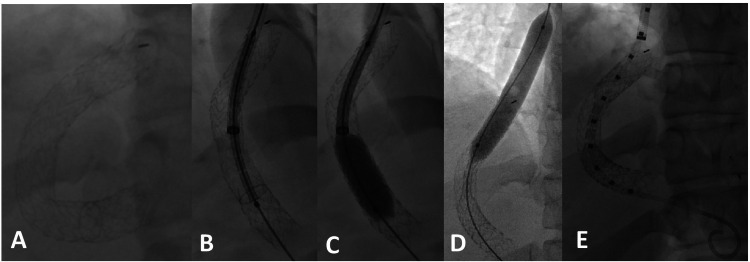
Single balloon-expandable covered stent technique for TIPS reduction TIPS reduction, single balloon-expandable covered stent technique. Image A demonstrates position and appearance of TIPS shunt prior to revision procedure. Alignment of nondeployed balloon-expandable covered stent (Viabahn VBX) within preexisting TIPS Stent (Viatorr) (B). Images C and D show balloon expansion of the hepatic and portal ends. “Waist” in the midsegment of the stent (E) allows a decreased volume of portal blood flow to bypass the liver while increasing hepatic portal perfusion. TIPS: transjugular intrahepatic portosystemic shunt.

In our unique method of TIPS reduction, a 10 x 59 mm Viabahn VBX balloon-expandable covered stent (W. L. Gore & Associates, Inc; Flagstaff, AZ) was delivered in coaxial fashion within the preexisting TIPS Viatorr stent under fluoroscopic guidance. During stent deployment and with the aid of a 10-French, 45-cm vascular sheath, the portal and hepatic ends of the VBX stent were dilated to 10 mm; yet, the midsegment of the stent was not dilated. This technique allowed for creation of a “waist” in the midsegment of the TIPS, thereby narrowing the lumen diameter and adequately constraining the TIPS (Figure 5A-5E). Effectively, this procedure increased the HVPG from 10 mmHg to 15 mmHg, consistent with the criteria for technically successful TIPS reduction described by Sarwar et al. [13], while maintaining patency of the TIPS shunt to prevent total shunt occlusion-related complications and recurrence of the esophageal varices. In comparison to the parallel stent technique described by Rowley et al. [12], our method only uses one stent rather than two stents to reduce the TIPS diameter, and this may be a more cost-effective method because it requires fewer stents. Our method may be more advantageous as it only requires one venous access site (typically the right internal jugular vein), rather than two venous access sites described by Rowley et al. [12] in the parallel technique. In the author’s opinion, deployment of a single covered stent as opposed to two separate stents allows for less error, procedure time, and potential procedural related complications. The VBX stent is balloon expandable, allowing better control during stent deployment to constrain the TIPS in contrast to the parallel technique. Lastly, the VBX stent method provides the option to readjust the shunt diameter if portal hypertensive complications arise in the future.

## Conclusions

HE is a common complication that arises in patients post-TIPS procedure. A notable portion of these patients may further experience HE refractory to pharmacological intervention and must be treated with invasive treatments such as TIPS reduction. The TIPS reduction procedure may result in a life-threatening recurrence of the initial TIPS indication. Therefore, it is imperative that clinicians balance the risks with the benefits of the procedure when treating refractory HE. Our case report presents a unique method of TIPS reduction using a single Viabahn VBX stent, which allows for procedural precision with fewer stents than other available techniques, and may serve as a quicker, safer, and more cost-effective option.
